# Wellbeing and Work Design in Brazilian Teleworkers

**DOI:** 10.3389/fpsyg.2021.733640

**Published:** 2021-10-21

**Authors:** Viviane Mishima-Santos, Marina Greghi Sticca, Amalia R. Pérez-Nebra

**Affiliations:** ^1^Laboratory of Organizational and Occupational Psychology, Department of Psychology, University of São Paulo, Ribeirão Preto, Brazil; ^2^Department of Administration, University of Brasília, Brasília, Brazil; ^3^Department of Psychology, Universidad Internacional de València, València, Spain

**Keywords:** work design, well-being, remote work, teleworker, work from home, work characteristics, multi-method, COVID-19

## Abstract

Studies suggest that work characteristics may be related to workers’ wellbeing. However, little is known about how these work characteristics may influence telework wellbeing in the face of the long period of social isolation and restrictions imposed by COVID-19. This study aimed to relate work characteristics in remote work to wellbeing using a two-stage multi-method approach. The general hypothesis is that different work characteristics will be organized into different groups and related to wellbeing. In Step 1, 108 teleworkers who participated in compulsory telework conditions answered the Work Design Questionnaire (WDQ) and Wellbeing at Work scale. A cluster analysis was conducted in which two clusters emerged based solely on their valence. The variables that contributed most to the cluster were: feedback from the job, social support, problem-solving, and decision and execution autonomy. Cluster 1 aggregated higher scores on work characteristics, and Cluster 2, lower scores. Cluster 1 presented significantly higher scores on wellbeing. In Step 2, 27 of these workers were blindly interviewed. Five classes of words emerged from the interviews: Class 1 – wellbeing, Class 2 – work dissatisfaction lexicon, Class 3 – role clarity, Class 4 – job demands, and Class 5 – job resources, including receiving feedback, conversations, praise, and support. Chi-square analysis suggests significant differences in classes 2, 3, 4, and 5. Cluster 1 appears more frequently in the role clarity class and less frequently in the work dissatisfaction and job demands classes. Cluster 2 is more frequent in the job dissatisfaction and job demands classes, however, less frequent in the job resources class. Class 1 shows no significant difference. These results partially support the general hypothesis that different work characteristics will be organized into different clusters and related to the teleworker’s wellbeing, but in the sense that it prevents suffering but does not necessarily promote wellbeing. The results contribute to the understanding of the relationship between work characteristics and wellbeing during the pandemic by using a different methodological approach, describing that work feedback, social support, skill variety, and problem-solving are the most significant in differentiating the perception of the groups. Social support and feedback from the job differentiate cluster 1 from cluster 2, but social support is not able to increase wellbeing, unless buffering unwellness.

## Introduction

Although studies based on work design models suggest relations between work characteristics and worker wellbeing (Morgeson and Humphrey, 2006; Parker et al., 2017; Montañez-Juan et al., 2019), there is less evidence particularly in the pandemic and teleworker field. Work design models have historically organized work characteristics in dimensions due to the shift in core work activity, from manufacturing economies to service and knowledge economies, that have dramatically altered the nature of work in organizations. However, little is known about how much these work characteristics can influence wellbeing in remote work in the face of the long period of social isolation and restrictions imposed by COVID-19 (Hodder, 2020; Kniffin et al., 2021; Ipsen et al., 2021; Wang et al., 2021), with pieces of evidence of both positive (Ipsen et al., 2021; Williams et al., 2021) and negative (Ipsen et al., 2021; Wang et al., 2021) repercussions.

The Covid-19 pandemic imposes the need for compulsory remote working in many parts of the world, but it does not affect all countries equally (Kniffin et al., 2021; Pérez-Nebra et al., 2021). In Brazil, a country that suffers more due to its political conduction and social inequalities, the practice of remote work and working from home were not widely used (Helliwell et al., 2021). Workers who had this opportunity had little remote work experience and their organizations were not prepared to support them. Recent data indicate that in 2018, 3.8 million people (less than 2%) performed their work activities in their households, a number that increased to 8.7 million after the onset of the pandemic in 2020 (Góes et al., 2020; IBGE, 2020).

In this sense, this work aimed to describe how employes perceive the characteristics of remote work, how these characteristics are organized, and their relationship with wellbeing. We also investigated the relation of work characteristics to wellbeing in remote work using a multi-method approach. Moreover, this study employed the term remote work as an umbrella term, including any employe who works outside of the traditional office and uses information and communications technology to access work (Grant et al., 2013).

The literature on work design and remote work identified three different approaches on how remote work could be related to wellbeing (Wang et al., 2021). In the first approach, remote work is an independent variable that predicts remote worker outcomes, and its impact on wellbeing depends on work characteristics as a moderator (Golden and Veiga, 2005; Golden et al., 2006; Perry et al., 2018). In the second approach, work characteristics act as mediators of the effect of remote work on outcomes, by changing job demands, autonomy, and relational aspects of work, which influence employe outcomes, such as work satisfaction (Gajendran and Harrison, 2007). Finally, in the third approach, work characteristics would be shaped by the telework context and directly influence the worker’s wellbeing. According to Wang et al. (2021), during the Covid-19 pandemic, remote work became the new context, and the third approach gained importance. Also, it is important to identify the best aspects of remote work associated with wellbeing, and we chose to adopt the last approach in this research.

The term work design is defined as the content and organization of the work tasks, activities, relationships, and responsibilities (Parker, 2014), and as the study, creation, and modification of the composition, content, structure, and environment in which jobs and roles are performed (Morgeson and Humphrey, 2008). While these definitions recognize the usefulness of work design for understanding how technology has affected several work contexts, they do not adapt the dimensions for the context of remote work where “unpredictability increases job complexity” (Kniffin et al., 2021, p. 71), increasing task cost and work intensification (Wang et al., 2021).

Wang et al. (2021) conducted an exploratory study of the direct relationship between work design and outcomes. Based on interviews, they described four core virtual work characteristics: social support, job autonomy, monitoring, and workload. However, it is a restricted approach to virtual work design. Morgeson and Humphrey (2006) offer a broad work design model that allowed descriptions of how much those work characteristics were present in the remote work.

Morgeson and Campion (2003) and Morgeson and Humphrey (2006) proposed a comprehensive set of work characteristics. The Work Design Questionnaire (WDQ) is an integrative set of 21 work characteristics, including the task, knowledge, social, and contextual domains. The task characteristics of the WDQ include autonomy (freedom to plan, decide, and implement work methods), task variety (need for multiple tasks), task significance (influence over other people’s lives), task identity (recognizable completed products), and feedback from job (getting direct and clear information on the effectiveness of task performance). Knowledge characteristics include job complexity (use of many higher-level intellectual skills), information processing (need to attend to and process data), problem-solving (need for unique ideas and solutions), skill variety (need for a variety of different skills), and specialization (need for knowledge and skills in a particular area). Social characteristics include social support (guidance and assistance from others), interdependence (dependence on others to complete the work, and dependence of others on the worker), interaction outside the organization (demand for interaction and communication with individuals external to the organization), and feedback from others (provision of information on performance by others in the organization). Finally, contextual characteristics, that *assumes* face-to-face work, includes ergonomics (provision of appropriate posture and movement), physical demands (level of physical activity or effort required), work conditions (presence of health risks; noise, temperature, and cleanliness of the environment), and equipment use (variety and complexity of the technology and equipment used). In this study, the contextual dimension will not be applied for two reasons, it is not adapted for remote work, and remote work is the context considered here. In this sense, questions emerge: Does confinement impact the perception of work characteristics, and do they decrease significantly (below the midpoint)?

Thus, the first hypothesis is:

The Covid-19 confinement influences the rating indices of the WDQ to be below the midpoint of the scale (H1).

Work design is recognized as a key antecedent of most dependent variables, including wellbeing/strain and job performance (Parker et al., 2017). Studies also pointed out that when work characteristics are positively designed, they generate wellbeing and performance (Humphrey et al., 2007). However, studies that relate wellbeing and work design usually describe the relation with one specific dimension of wellbeing, namely job satisfaction.

The relationship between work design and wellbeing was explained by several models that in general consider two opposite types of work characteristics. Those which were negative, demands, and those which were positive, as a resource [i.e., Job Demand-Resource model (JD-R) from Bakker and Demerouti (2017)], or job decision latitude (Karasek, 1979). Job demands are defined as psychological, physical, social, or organizational aspects of the job that require human or organizational cost (Demerouti et al., 2001). Examples of job demands are: high work pressure and emotionally demanding interactions. Job resources refer to those functional characteristics in achieving work goals, reducing job demands, and stimulating personal growth, learning, and development (Bakker and Demerouti, 2007). Examples of job resources are autonomy, skill variety, performance feedback, and growth opportunities. Although it is possible to describe some typical examples of demands and resources, some work characteristics are not evident, for instance, job complexity or information processing, which for some people could be interpreted as a challenging and exciting job (challenge stressor), and for others could be interpreted as a high-cost activity (hindrance stressor) (Crawford et al., 2010).

Regarding wellbeing, two distinct philosophical streams guide studies on the field, the hedonic approach and the eudaimonic approach (Ryan and Deci, 2001). The latter consists of positive subjective states (Waterman et al., 2008) and for the hedonic approach, wellbeing is an experience of feeling good. This view has two main orientations, the emotional (e.g., emotions and affections) and the cognitive (e.g., satisfaction) (Peiró et al., 2021). For eudaimonism, wellbeing is an experience of fulfillment and purpose, self-realization, the search for meaning in life (Waterman, 1993; Ryff and Keys, 1995; Diener, 2000; Sonnentag, 2015). In this case, there are two other eudaimonic orientations, one for the present (e.g., engagement in work) and one for future orientation (e.g., meaning of work) (Peiró et al., 2021).

The wellbeing scope could be broad, but in the present case, remote work in the pandemic time, it is possible to advocate for two broad dimensions of wellbeing, one hedonic and the other eudaimonic. The most typical wellbeing measure is job satisfaction, and it was found related to work characteristics in the context of remote work (Wang et al., 2021). Although job satisfaction is a cognitive orientation, it is domain-specific, includes different facets, and is more stable than emotions. Job satisfaction is “the degree to which a person reports satisfaction with intrinsic and extrinsic features of the job” (Warr et al., 1979, p. 133). As an eudaimonic variable, the sense of connection and involvement with your work in the pandemic period is valorized, perhaps needed.

In the JD-R, Bakker and Demerouti (2017) suggested that the resources are instigated by a motivational process that includes work engagement and organizational commitment. Work engagement, a eudaimonic wellbeing concept, is “the mental state where employes feel full with physical energy (vigor), are enthusiastic about the content of their work and the things they do (dedication), and are so immersed in their work activities that time seems to fly (absorption)” (Bakker and Demerouti, 2017, p. 2). Organizational commitment, however, is not a wellbeing measure, but it is included in some models as such (e.g., Siqueira and Padovam, 2008; Siqueira et al., 2014), it shares similarities depending on how it has been conceptualized. For instance, in some definitions, it is similar to engagement, as “the relative strength of an individual’s identification with and involvement in a particular organization” (Mowday et al., 1979, p. 226) or that high scores on the scale reflects affective attachment with colleagues and the organization (Allen and Meyer, 1990; Meyer and Allen, 1991). The combination of work engagement and organizational commitment could be favorable because both, according to Bakker and Demerouti (2017), could have a positive role in wellbeing.

Returning to the relationship between work design and wellbeing, work design is composed of work characteristics that are organized into four dimensions, three of which generally have empirical evidence of a relationship with wellbeing: task characteristics, knowledge characteristics, and social characteristics (Morgeson and Humphrey, 2006; Stegmann et al., 2010; Bigot et al., 2014; Montañez-Juan et al., 2019; Pérez-Nebra et al., 2020). The exceptions are some social work characteristics, such as interdependence (initiated or received) and interaction outside the organization, which are not related to wellbeing (Stegmann et al., 2010; Bigot et al., 2014). Most task characteristics, such as autonomy, other job resources (i.e., job control, feedback, and task variety), and opportunities for learning and development, are mainly linked to positive changes in wellbeing over time that translate more into an increase in work engagement and other positive indicators of wellbeing rather than a decrease in negative indicators (Sonnentag, 2015). Although we found evidence from the same dimension, i.e., social characteristics, both related and not related to wellbeing, this does not mean that the work characteristics will be grouped accordingly, and there is also evidence from the group of characteristics related to wellbeing (e.g., Montañez-Juan et al., 2019). In this sense, are the work characteristics, organized initially into different dimensions, going to be organized in the same way in a remote work context?

Thus, the second hypothesis of this work is: The data will be organized into three clusters based on the dimensions of the WDQ (task characteristics, social characteristics, and knowledge) based on the Work Design theory proposed by Morgeson and Campion (2003) and Morgeson and Humphrey (2006) (H2).

In remote work, the impact on wellbeing also depends on other work characteristics, including task interdependence (Golden and Veiga, 2005) and job autonomy (Perry et al., 2018). Studies also have examined how social and interpersonal factors predict changes in wellbeing over time, addressing social support, negative social interactions, and leadership processes as possible predictors (Wang et al., 2021). Bentley et al. (2016), in a study on organizational support in the wellbeing of teleworkers, argue that organizational social support improves workers’ wellbeing. When organizational support is present and effective, it is positive for wellbeing, however, when this support is non-existent or late, the worker feels pressured, unsupported, and negatively impacting wellbeing (Lapierre and Allen, 2006; Vander Elst et al., 2017; Wang et al., 2021).

Results from studies on remote work in pandemics support similar results in non-pandemic contexts. Thus, it is possible that virtual work characteristics may improve the effectiveness and wellbeing of remote workers (Wang et al., 2021). The results in the remote work context pointed out that social support and job autonomy, acting as job resources, help employes cope with the challenges of remote work, workload, and monitoring (Wang et al., 2021). Similar results were found in different contexts where social support and job autonomy were found to be positively related to job satisfaction or wellbeing (Morgeson and Humphrey, 2006; Stegmann et al., 2010; Bigot et al., 2014; Pérez-Nebra et al., 2020). However, other work characteristics were found to be negatively related to job satisfaction, such as job complexity (Morgeson and Humphrey, 2006; Stegmann et al., 2010) which could work as a demand and was not found in the pandemic context.

In the case of social support it is important to point out that although it is a work characteristic, it is also a coping strategy, and integrated into the job demand-control model to predict wellbeing (Johnson and Hall, 1988). In general, studies attest that social support reduces psychological distress during stressful times (Taylor, 2010). However, not all studies show beneficial effects of social support (Taylor, 2010) and particularly in Brazil, it was found that social support increases stress responses to remote workers (de Almeida Fonseca and Pérez-Nebra, 2012). Thus, in the pandemic context, where the structural support and the number of social relationships decreases, it is possible to think that social support could act as a resource or a demand. Therefore, social support can increase remote workers’ work-home interference and, thereby, negatively or positively affect their wellbeing. Social support seems to be the most powerful remote work characteristic because it had positive indirect impacts on performance and wellbeing (Wang et al., 2021). Hence, the other research question is related to the intertwining between work characteristics and the wellbeing described by workers. Will different work characteristics emerge in different reports of wellbeing?

The third hypothesis of this work is: Clusters with high scores on the task and social dimensions will have more positive reports of wellbeing (H3).

This study highlighted a significant gap in understanding how work characteristics can be related to wellbeing in telework. Given the long period of social isolation and restriction imposed by COVID-19, in a country like Brazil, which is the sixth-largest country in terms of population, where nearly 55 million people earn less than €80 a month and thus live below the poverty line (IBGE, 2018; Pérez-Nebra et al., 2021), remote work takes on different configurations based on political and poverty issues (Helliwell et al., 2021). Thus, remote working is done by a privileged minority, those who earn higher-incomes, highly educated, and white-collar workers (Pérez-Nebra et al., 2021). Individual wellbeing cannot be separated from social wellbeing, the privileged wellbeing may occur at the expense of the wellbeing of others in this context (Pérez-Nebra et al., 2021). In other words, upward and downward social comparison occurs in the remote worker context (Diener et al., 1999). We contributed to the literature by testing clusters of work characteristics and describing which is more central to differentiate clusters. We also offered a multi-method methodological approach of work characteristics and wellbeing, where we could describe other facets of this relation.

## Materials and Methods

### Study Design and Procedures

The Research Ethics Committee of the University of São Paulo approved this study under number 03080718.1.0000.5407. We used a two-step multi-method procedure. In Step 1, we used a quantitative approach to examine the Work Design and Wellbeing at work validated for Brazil with good psychometric qualities.

In Step 2, a qualitative approach was used to examine the relationship between work characteristics and wellbeing. Semi-structured interviews were applied to have deep and unique content related to wellbeing in the compulsory telework condition. We used an interview script based on the work design and psychological wellbeing model validated by judges. This meant that, in practice, each interview involved researchers and participants engaging in an informal, casual conversation exploring each participant’s personal experience (Bhattacharya, 2017) concerning telework and wellbeing.

The focal questions included in the interview roadmap explored (1) task characteristics and wellbeing (e.g.,: How is autonomy related to your wellbeing?) (2) knowledge characteristics and wellbeing (e.g.,: How is the degree of complexity related to your wellbeing?) (3) social characteristics and wellbeing (e.g.,: How are labor relations related to your wellbeing?).

Participants were provided with information about the study, and provided written consent before the interviews were conducted. Individuals stated their age, sex, and type of telework on the consent form to report on the demographic details of the sample. All interviews were conducted online, were recorded, and completely transcripted.

### Sample

Participants were recruited from Internet forum posts and advertising. The researchers accessed different teleworker forums, such as Instagram, Whatsapp groups, and Brazilian forums on telework. The research was presented, and a hyperlink to the questionnaire was provided. This strategy was adopted to reach a diversified public of teleworkers.

Data were collected online (questionnaire *via* hyperlink and interviews were *via* Google Forms). In Step 1, 108 Brazilian remote workers participated in the survey, 45 (41.7%) were independent professionals, 41 (38.0%) of whom were employed, 20 (18.5%) self-employed teleworkers, one (0.9%) entrepreneur, and one (0.9%) fixed term contract, with a mean age of 36.63 years (SD = 8.75).

In Step 2, 27 teleworkers participated in the survey. Although we had initially made a cluster balanced interviews list, expecting negatives from respondents, 17 from Cluster 1 and 10 from Cluster 2 accepted to take the interview. They were a subsample of Step 1, including 9 women (33.33%) and 18 men (66.67%) with a mean age of 35.75 years (SD = 8.53), belonging to different areas of activity, including IT professionals, entrepreneurs, pharmaceutical industry managers, consultants, digital marketing specialists, lawyers, and others with different employment relationships (employed, self-employed, freelance, and legal person). Participants worked in private and third sector companies. Of these, 23 (85.19%) reported that they performed activities in home-office, the best-known variant practiced by professionals in remote work, and 4 (14.81%) performed the activity in hybrid mode (home office and company office). It is important to point out that all the 27 professionals in the sample were already engaged in the teleworking modality before the pandemic.

### Measures

#### Work Design

We used Morgeson and Humphrey’s (2006) model with three general dimensions of work characteristics: task, knowledge, and social. We used the scale adapted to the Brazilian population (Borges-Andrade et al., 2019) and selected the task, knowledge, and social dimensions. As pointed out, we decided not to measure work context because the scale was built for a context where people are working together in a shared place, and not adapted for the remote work context. Therefore, the response options consisted of a five-point scale from 1 (totally disagree) to 5 (totally agree).

To measure task characteristics, we had work planning autonomy (3 items, Crombach’s α = 0.83), decision and execution autonomy (6 items, α = 0.94), task variety (4 items, α = 0.94), task significance (4 items, α = 0.79), task identity (4 items, α = 0.90), and feedback from job (3 items, α = 0.92). Knowledge characteristics included job complexity (3 items, α = 0.82), information processing (4 items, α = 0.80), problem-solving (6 items, α = 0.85), and specialization (4 items, α = 0.76). Finally, social characteristics included social support (6 items, α = 0.87), interdependence (5 items, α = 0.87), interaction outside the organization (4 items, α = 0.91), and feedback from others (3 items, α = 0.92).

#### Wellbeing at Work

We applied the Siqueira et al. (2014) version of wellbeing at work scale^1^. Although the scale is composed of three factors, job satisfaction (e.g., I am satisfied with the degree of interest that my tasks arouse in me), engagement (I am interested in the organization where I work), and commitment (I am proud of the company I work for), for parsimony we used it as one general factor of wellbeing (13 items, α = 0.95). Participants were asked to rate each item on a 5-point scale, ranging from 1 (Totally disagree) to 5 (Totally agree), considering the agreement with each statement based on their current work.

#### Control Variables

We controlled sex (0 = male, 1 = female), age in years, the state of Brazil, where the person works, type of contract (autonomous or employed), seniority in the organization, seniority at work, number of years the person has worked from home, and size of the organization. We considered that these variables could be a significant contribution to the difference in work design.

#### Interview Script

A semi-structured interview script was developed based on the dimensions of the WDQ (Morgeson and Humphrey, 2006), relating the questions to wellbeing. At this stage, a content validation was performed with four expert judges in the field who received the digital document and provided feedback on the questions’ semantic validation, quality, and study purpose. The judges asked to include a definition of wellbeing to facilitate understanding of issues and changes in the format of some questions to avoid laconic answers. We also conducted a pilot study with seven teleworkers to verify the remote workers’ understanding and comprehension of the terminologies and the clarity of the questions. Changes were made to broader ones (e.g.,: we changed the term telework to remote work) and allowed the inclusion of independent professionals’ reality (e.g.,: “do you receive support from customers, family, friends, people outside your work?”), since the questions were focused on employed workers (e. g.: “do you receive support from your manager and co-workers?”).

### Data Collection

#### Step 1: Questionnaire

In stage 1, a structured questionnaire was applied in two parts, the first on sociodemographic and labor data, and the second on the WDQ and Wellbeing at work. In this form, we asked those who wished to answer a second stage of the project to provide an email address for further contact. The dataset is disponible in Santos et al. (2021).

#### Step 2: Interviews

In Step 2, the interviews were conducted blindly by a single researcher, that is, a draw was made to choose the sample to be interviewed out of the 108 questionnaires applied. Not all candidates agreed to participate in this stage of the study and a total of 27 interviews were conducted. This stage consisted of a double-blind study to avoid inducing the researcher to the data collected. We call double-blind the fact that the interviewer, when given the list of which interviews to perform, did not know which cluster the interviewee belonged to.

The interviews were conducted online *via* Google Meet, and the interview script was previously sent *via* email to each participant before conducting the interviews, to facilitate their approach and preparation. All participants in the survey were invited for an interview. We send a formal invitation *via* e-mail. Only 27 workers agreed in realize the interview and constituted the final sample. In Step 2, with the participants’ consent, all interviews were audio-recorded. Each recording was transcribed with the help of Otranscribe and MXQDA, version 20.2.1 to streamline the process and verify the total reliability of the information collected.

### Data Analysis

#### Preliminary Analysis

The study showed a minimal percentage among the missing values (up to 1% in demographic characteristics, 0% in the work design and wellbeing scale). We conducted reliability and descriptive and correlation analyses before performing the cluster analysis with the work design variables. The whole scale presents acceptable reliability. The correlations were below 0.30 on average. Next, we conducted the subsequent step of the analysis.

#### Cluster Analysis

The 108 workers were clustered based on their individual level of work design to identify relationship patterns, using a two-step procedure in the SPSS. The log-likelihood distance measured the distance between the 14 variables. Since the correlations between the work design variables were weak, we opted for the log-likelihood method. The log-likelihood distance measure used in the two-step cluster assumes that the variables are normally distributed and independent (Kent et al., 2014). The two-step procedure combines hierarchical and non-hierarchical methods, forming two clusters efficiently. Descriptive statistics and comparison means were conducted to get an accurate picture of the clusters.

#### Lexical Analysis

We transcribed all the interviews literally. After the transcription, we organized the corpus. We did it by standardizing the Portuguese language and connecting keywords. For example, wellbeing had to be rewritten as well_being. Other words have considerable differences in meanings and the same spelling, such as “*legal*,” which means legal/law in English and great/fine/cool in Brazilian Portuguese.

The lexical analysis was conducted using the Iramuteq software and the Camargo and Justo (2018) Iramuteq protocol. We conducted 27 interviews, with 2,125 segments, 74,153 occurrences, and 46.75% of hapax. We also conducted Reinert Classification with Descendent Hierarchical Classification (DHC) and Correspondence Factor Analysis (CFA).

#### Lexical Analysis Comparison

To compare the classes of the lexical analysis with both clusters, we conducted a chi-square analysis. This analysis allowed us to identify the differences between groups. It is a similarity analysis carried out on an absence/presence of the group which crosses the selected units in a row and the active forms of the class in a column. Those differences were considered significant when the test is greater than 3.84, based on 1 degree of freedom and *p* < 0.05.

## Results

The descriptive results (Table 1) suggest that, in general, the sample presented a high quality of work design, as the averages were above the medium point of the scale. Furthermore, unlike what was posited in Hypothesis 1, all the work characteristics measured were above the scale’s central point. In general, the relation between work characteristics are positive. However, it is not possible to affirm that the work characteristics that compose each dimension have a more intense relation between them, which possibly impacts the cluster analysis in the sense that the cluster may differ in how it will be composed. Negative relations are with job complexity and information processing, in this sense, these variables could be assumed as being demanding characteristics for this sample. Moreover, in general, wellbeing is positively related to all work characteristics, except for job complexity.

**TABLE 1:** Descriptive, correlations and Cronbach’s alpha on the diagonal.

	M (SD)	1	2	3	4	5	6	7	8	9	10	11	12	13	14	15
(1) Work planning autonomy	4.27 (0.86)	(0.83)														
(2) Decision and execution autonomy	4.09 (0.93)	0.75**	(0.79)													
(3) Task variety	4.31 (0.88)	0.05	0.28**	(0.94)												
(4) Task significance	4.42 (0.66)	0.35**	0.46**	0.13	(0.79)											
(5) Task identity	4.13 (0.85)	0.28**	0.21*	−0.02	0.33**	(0.90)										
(6) Feedback from job	3.80 (1.01)	0.28**	0.31**	0.11	0.43**	0.39**	(0.92)									
(7) Job complexity	3.52 (0.94)	−0.21*	−0.19*	0.17	−0.14	−0.22*	−0.18	(0.82)								
(8) Information processing	4.43 (0.63)	−0.11	0.10	0.49**	0.11	−0.09	0.20*	0.27**	(0.80)							
(9) Problem-solving	4.34 (0.63)	0.05	0.26**	0.46**	0.31**	0.01	0.18	0.19	0.53**	(0.85)						
(10) Specialization	4.08 (0.75)	0.06	0.18	0.21*	0.26**	0.28**	0.33**	0.14	0.29**	0.42**	(0.76)					
(11) Social support	4.02 (0.80)	0.20*	0.32**	0.46**	0.36**	0.10	0.30**	0.01	0.27**	0.36**	0.06	(0.87)				
(12) Interdependence	3.30 (1.05)	0.09	0.08	0.04	0.03	−0.04	0.04	−0.10	0.09	0.09	0.28**	0.14	(0.87)			
(13) Interaction outside the organization	3.71 (1.13)	0.17	0.22*	0.24*	0.12	−0.01	0.13	−0.07	0.23*	0.28**	0.12	0.29**	−0.03	(0.91)		
(14) Feedback from others	3.35 (1.14)	0.18	0.28**	0.18	0.32**	0.15	0.50**	−0.10	0.20*	0.27**	0.22*	0.45**	0.21*	0.05	(0.92)	
(15) Wellbeing	3.94 (1.03)	0.37**	0.42**	0.21	0.48**	0.38**	0.45**	−0.21	0.10	0.28**	0.10	0.50**	0.05	0.03	0.64**	(0.95)

*p < 0.05; **p < 0.01.

A two-cluster solution was identified in the two-step cluster analysis. Cluster 1 aggregates 65.74% (*N* = 71) of the sample and Cluster 2, 34.26% (*N* = 37) of the sample. Silhouette is considered fair (3) (Rousseeuw, 1987). This result refutes Hypothesis 2 of the study, which posits a 3-cluster solution from the dimensions proposed in the WDQ. Table 2 shows the importance of the mean of the cluster predictor, and the test of differences of means. It can be observed that the cluster was organized based on valence and not on the work design dimensions. Regarding contribution, on the one hand, the work characteristics that most contribute to the clusters are Feedback from job, problem-solving, and social support. On the other hand, the variable that least contributes to the cluster is job complexity. In this sense, even being a demanding variable, it does not contribute to differentiate the clusters.

**TABLE 2:** Descriptive and differences between Cluster 1 (*N* = 71) and Cluster 2 (*N* = 37).

	Cluster 1	Cluster 2	Significance	*Z*
			
	M (SD)	M (SD)		
Work planning autonomy	4.53 (0.57)	3.79 (1.10)	0.54	21.05***
Decision and execution autonomy	4.45 (0.54)	3.42 (1.15)	0.90	39.73***
Task variety	4.56 (0.64)	3.86 (1.09)	0.46	17.53***
Task significance	4.64 (0.46)	4.01 (0.78)	0.68	28.16***
Task identity	4.21 (0.80)	4.00 (0.93)	0.07	1.52
Feedback from job	4.19 (0.76)	3.04 (0.98)	1.00	45.71***
Job complexity	3.55 (0.98)	3.46 (0.87)	0.02	0.24
Information processing	4.64 (0.45)	4.04 (0.72)	0.66	27.06***
Problem-solving	4.58 (0.42)	3.89 (0.70)	0.91	40.55***
Specialization	4.31 (0.67)	3.62 (0.71)	0.61	24.72***
Social support	4.52 (0.48)	3.62 (0.98)	0.91	40.64***
Interdependence	3.48 (1.04)	2.95 (1.00)	0.21	6.60**
Interaction outside the organization	3.91 (1.05)	3.34 (1.19)	0.21	6.50*
Feedback from others	3.75 (0.92)	2.58 (1.14)	0.77	32.93***

*p < 0.05; **p < 0.01; and ***p < 0.001.

We compared control variables between the clusters and wellbeing (Table 3). As can be seen, no control variable showed a significant difference. Nevertheless, wellbeing was a significant difference, wherein Cluster 1, with better work conditions, shows better wellbeing when compared to Cluster 2. Cluster 2 presented slightly more women, but the difference is non-significant for this sample.

**TABLE 3:** Test control variables between clusters.

Variable	Cluster 1	Cluster 2	*Z*
Sex (men = 1; women = 2)	1.39 (0.49)	1.54 (0.51)	2.11
Age	37.37 (8.65)	35.21 (8.87)	1.48
Seniority in the organization	5.31 (6.40)	5.59 (5.31)	0.05
Time in working from home	5.03 (4.71)	4.52 (5.78)	0.25
Seniority	14.63 (9.73)	13.43 (8.88)	0.39
Manager (1 = no; 2 = yes)	1.56 (0.50)	1.51 (0.51)	0.24
Wellbeing	4.16 (0.93)	3.48 (1.03)	8.70*

*p < 0.01.

**FIGURE 1:**
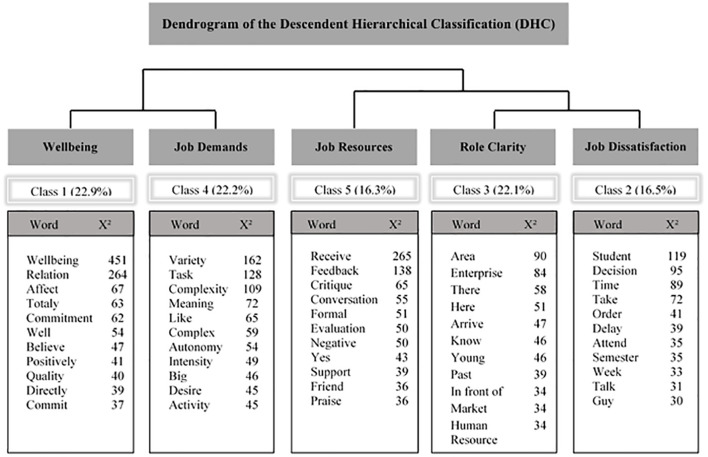
Dendrogram of the Descendent Hierarchical Classification (DHC).

The DHC organized five classes of words. They emerged from the interviews of which two couples of variables are related, classes 1 and 4, and 2 and 3. Class 1 we named “Wellbeing” as the most frequent word is wellbeing. We named Class 4 “Job Demands” because the lexicons that emerged are related to job complexity and task variety. Those work characteristics are negatively or lowly related to wellbeing (cf. Table 1). Class 2, “Job Dissatisfaction,” more evident from the speech extractions than the lexicons, aggregates complaints and problems related to work. Class 3, “Role Clarity,” again, more evident from the extracts of speech than from the lexicons, aggregates knowledge about the task, how human resources give explicit norms of what is expected from them. Class 5, “Job Resources,” differs from all others. It consists of lexicons on job resources, such as receiving feedback, conversations, praise, and support (Figure 1).

Table 4 represents some examples of representative speeches for each category. Iramuteq provides these verbal accounts. They contain a weighting, in descending order, of the most representative utterances for each word class. The most typical reports are presented here for illustration. These reports, combined with the lexicals, helped us to name each lexical class.

**TABLE 4:** Speeches related to each class.

Classes	Examples of speech
(1) Wellbeing	“I think the feedback is very important and it is well related to wellbeing” “Autonomy is totally related to my wellbeing, 1,000 percent related, it’s very important to me” “I really like having autonomy, having control over my activities, over my schedules and everything else, and as I like having autonomy, it’s 100 percent related to my wellbeing, I’m very happy”
(2) Work dissatisfaction	“First, to change my schedule I need to consult the student…until the student answers me, it’s an agony, especially if I have another class I’ll have to change because then it becomes a domino effect” “And sometimes we have to make a decision that is not nice, for example, sometimes we have to disconnect someone from the company”
(3) Role clarity	“Of course the experience helps a lot because we already have a certain amount of time in the market, but the degree of complexity is great, our work varies, I do a little of everything, I know the whole process” “For me, this variety that we experience today in XXX is good because we know it from all fields within the area”
(4) Job demands	“I feel good, the degree of variety is high, as I said there are several varied tasks that happen on the same day, maybe different excitements, so it’s a high degree of variety” “The degree of variety is something I like, new challenges every day and doing different things.”
(5) Job resource	“I have constantly received feedback on tasks in formal ways, when it comes to my manager we have feedback routines that we conduct” “I get general support, both emotional and acknowledging how good my performance is”

**FIGURE 2:**
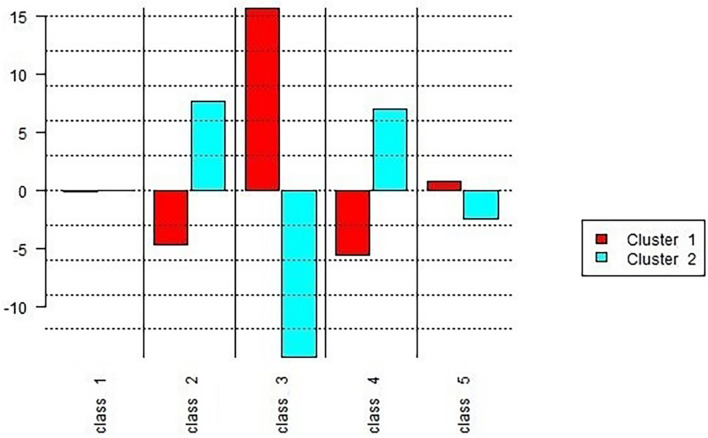
Chi-square between clusters and classes of lexicons.

The chi-square analysis suggests significant differences in classes 2, 3, 4, and 5 (Figure 2). Cluster 2 appears more frequently in classes 2 and 4, and Class 5 appears least in Cluster 2. Cluster 1 appears more frequently in Class 3. Class 1 showed no significant differences. Hypothesis 4 assumed that higher work characteristics scores were related to more positive verbalizations about work from home. Class 1 showed more words related to wellbeing and showed no difference refuting the hypothesis. However, the classes that present differences align with the hypothesis. Role clarity (Class 3), where speeches of Cluster 1 are more frequent, is related to greater work design. Also, Cluster 2, with lower work characteristics, is more frequent in Classes 2 and 4. Class 2 is related to feeling disrespected and job dissatisfaction. An example of speech is “I sent a message and the student told me at the last minute that he couldn’t come.” Class 4 is related to job demands; for example, “Sometimes it really gets in the way, but it happens. I think it is very much part of my job to have this great variability”; and Cluster 2 shows fewer speeches of Class 5 that aggregate lexicon of job resources. Overall, these results suggest that the low work characteristics are related to illbeing (e.g., work dissatisfaction), but the higher work characteristics are only related to a wellbeing dimension. The lack of resources and increased demands are also more frequent in the low work characteristic group (Cluster 2).

## Discussion

This study aimed to relate the work characteristics to wellbeing in remote working using a multi-method approach. H1 posited that confinement would impact the perception of work characteristics and these were notably lower, below the midpoint, so it was not confirmed. H2 suggested that the data would be organized into three clusters from the Work Design dimensions (task, social, and knowledge characteristics), and it was not confirmed either. Finally, as H2 was not supported, the clusters were organized by its valence and not by its dimension, H3 suggested that the clusters would be more frequently related to wellbeing and was partially supported since the most positive clusters were related in the wellbeing at work scale scores and verbally in interview analyses.

As for the fact that the work characteristics were presented above the midpoint of the scale, albeit unexpected, there are some explanatory possibilities. The first explanation for this result may be the evaluative bias of the respondents, who in this case had telecommuted before, which may have led to a positive evaluation due to previous experiences. Another explanation is based on the context of the Covid-19 pandemic in Brazil and the unemployment scenario, which may have led to positive evaluations related to the fear of being unemployed and/or insecurity. The effect of social comparison on wellbeing is described in natural settings, mainly the upward and downward comparison (e.g., in the current case, comparing oneself having a safe work to someone that not even work has) being typically associated with higher subjective wellbeing than upward comparisons (e.g., comparing oneself having an intensification and accelerated work to someone in a more easy-going situation) (Diener et al., 1999). Finally, another explanation already found in longitudinal attitudinal studies is that evaluations do not vary as much as we might believe, even in changing scenarios. They may change dynamically, but they also stabilize quickly (e.g., Staw, 1986).

The work characteristics organization by valence rather than by dimension of the work project was also not expected, but it is not especially a novelty. One explanation for such a result is that workers evaluate the work project in general. In this case, the evaluation occurs more or less negatively concerning all aspects of the work, rather than by evaluating each dimension separately. Finally, another question to ask is whether work characteristics have become less central in terms of importance in this pandemic context in Brazil, when comparing having a job or not, with the opportunity to be safe at home, and so there was an “overall” evaluation.

Regarding the organization of clusters, it is important to highlight that a characteristic of the work of each dimension of the work design contributed the most to the organization of the clusters, namely: work feedback, social support, skill variety, and problem-solving. Such data are in agreement with previous studies conducted during the Covid-19 pandemic that also pointed out the importance of autonomy and social support, which when functioning as job resources, help professionals deal with the challenges of remote work (Wang et al., 2021), but also outside the context (Morgeson and Humphrey, 2006; Stegmann et al., 2010; Bigot et al., 2014). Moreover, they are consistent with the results, as work feedback is possible when there is also clarity regarding work and roles, which is the description of Class 3. In this sense, these results support previous studies (Wang et al., 2021).

H3 suggested that different groups would have different lexicons. In this sense, this study supports this hypothesis. However, the clusters were organized into positive and negative features, and the positive cluster (Cluster 1) was more frequent in the class of role clarity and less frequent in the negative features, such as job dissatisfaction and job demands. It is worth noting that Cluster 1 was not more prevalent in the speech about wellbeing or resources, as one might expect. The negative cluster (Cluster 2), on the other hand, was more frequent in dissatisfaction at work and perception of demands, and less frequent in perceived resources, i.e., it seems to be more related to absence, such as an expectation that was not met. It is noteworthy that the fact that the lexicons “like” followed by “autonomy” and “meaning” emerged among the class of job demands and were related to the cluster with low levels of work characteristics. There are some possible explanations for this finding. The first is social desirability; some employes could describe that they “like complexity” because it could be interpreted as trendy. Moreover, as this class is related to the Wellbeing class, the alternative explanation is that this class is a kind of Job Resource but as it was related to the low work design cluster, it could be interpreted that even though it qualifies as a challenging and positive resource, it drains. Another possible explanation is that this class could be interpreted as a personal resource (e.g., job crafting); however, if it is the case, this sort of resource is independent (in fact, opposite) to job resource (Class 5) in the qualitative analysis (the CFA suggest it^2^). Thus, these results suggest that the work characteristics of telecommuting in the context of the Covid-19 pandemic were able to “de-emphasize” the perception of unwellness. However, they were not able to increase wellbeing, i.e., they prevent unwellness, but do not promote wellbeing.

Still related to the combined results, it is important to note that the demand and resource model emerges. In this case, demand more clearly differentiates the groups than resource, where only its absence is evidenced and both related to the malaise. Considering that the demand and resource model is for burnout reduction (Demerouti et al., 2001; Bakker and Demerouti, 2007, 2017), it is consistent with the results found here. One of the contributions of this work was to understand the role of other variables in this remote context that are able to increase wellbeing (and not only avoid discomfort), such as role clarity and feedback from others.

Another point to be highlighted is that the perceived resource refers to emotional and informational or instrumental social support. Social support and feedback from others were the dimensions that most differentiated clusters 1 and 2. Moreover, the discourses referring to resources are fundamentally focused on feedback and supportive relationships. Thus, it is possible that emotional social support, such as it was found in other remote work contexts (de Almeida Fonseca and Pérez-Nebra, 2012), is functioning only as a way to disperse the unwellness and not actually able to increase wellbeing as one would expect, similarly to what occurred with the present sample.

### Limitations and Future Directions

This work is not without limitations. First, regarding the topics inherent to the research methods used, the data were collected in Brazil, which may raise concerns about generalizability. The study also has limitations related to the method (two-step procedure) used, which is constrained to the sample analyzed. The entire sample was composed of remote workers who already worked remotely before the pandemic and from which they had adapted “on the fly”; thus, findings cannot be generalized to the country level or strictly for new-remote workers. However, it is important to point out that there was no significant difference in time in working from home variables between clusters. Also, the sample comprises workers of higher socioeconomic levels and higher education levels, variables that allow them to perform home office work. Remote work in Brazil and developing countries is new and less widespread, and further studies that follow the process longitudinally are needed. Thus, it will be interesting to compare remote working during the pandemic between developing and developed countries with cross-cultural samples to analyze these findings’ generalizability and how cultural factors shape the impacts of virtual work characteristics on other remote worker outcomes.

Another limitation was the absence of the contextual dimension. The context in which the telework is conducted is relevant and describing the kind of context that could improve their wellbeing is a relevant question that was not achieved in this work. It is important to develop instruments of contextual work characteristics related to remote work^3^.

From a practical point of view, this work is believed to have implications for helping organizations and leaders to manage remote work effectively. First, the work design approach helps managers to consider how remote work can be designed to achieve wellbeing by focusing on setting clear goals, favoring clear tasks and identification with the task, and providing measures that allow for feedback on one’s work so the worker can perform a self-assessment and these factors can lead to wellbeing in remote work.

In addition, feedback appears as an important remote work characteristic. In this sense, future studies could analyze whether feedback is a central factor in remote work, in general, or whether it assumes greater importance in the context of the pandemic. As for the emergence of social support as a differentiating factor in remote work, Wang et al. (2021) found similar results in a different Brazilian context, so managers and human resource practices should favor supportive practices in the context of the Covid-19 pandemic and the use of hybrid work models, aiming to build a climate of trust and organizational civility, and share clear information of what is expected, rather than monitoring and controlling work, for example, in addition to other ways of interaction between teams and managers to take place. As a research agenda, we suggest studying other professional categories working from home at a higher physical or emotional cost, in precarious conditions of family or work support, and differentiate challenges and hindrance job demands, and personal and job resources in the remote work context.

## Conclusion

As initially pointed out, the present results suggest that work characteristics (work feedback, social support, skill variety, and problem-solving) affect the wellbeing of workers in telework. Social support and feedback from others were the dimensions that most differentiated clusters and role clarity is the most favorable point in increasing work characteristics. Through the use of a multi-method procedure it was possible to broaden the comprehension of the relationship between work characteristics and wellbeing in remote work by identifying in the qualitative analyses that emotional social support is not able to increase wellbeing, but to act as buffering unwellness.

The classic features of the work design proposed as a resource and demand in other studies did not contribute to the differentiation of the clusters. Finally, this work can be used as a basis for redesigning the characteristics of remote work by applying both preventive (e.g., using people management practices aimed at supporting employes) and therapeutic interventions (e.g., anticipating changes in public policies), and offers a new perspective in favor of a healthier work environment.

## Data Availability Statement

The datasets generated for this study can be found at Mendeley Data: doi: 10.17632/srwxkrr7vm.1.

## Ethics Statement

The studies involving human participants were reviewed and approved by the Research Ethics Committee of the University of São Paulo approved this study under number 03080718.1.0000.5407. The patients/participants provided their written informed consent to participate in this study.

## Author Contributions

MS and AP-N: conceptualization. VM-S: data curation, funding acquisition, investigation, and resources. AP-N: formal analysis, validation, visualization, and writing – review and editing. VM-S, MS, and AP-N: methodology. MS: project administration and supervision. VM-S and MS: writing – original draft. All authors have read and agreed to the published version of the manuscript.

## Conflict of Interest

The authors declare that the research was conducted in the absence of any commercial or financial relationships that could be construed as a potential conflict of interest.

## Publisher’s Note

All claims expressed in this article are solely those of the authors and do not necessarily represent those of their affiliated organizations, or those of the publisher, the editors and the reviewers. Any product that may be evaluated in this article, or claim that may be made by its manufacturer, is not guaranteed or endorsed by the publisher.

## Footnotes

1 The authors would like to thank the reviewers for this inclusion.

2 As this result is in Portuguese we did not present it, but it can be demanded to the authors.

3 The authors would like to thank the reviewers for this important point.
